# Exosomes Mediate Hippocampal and Cortical Neuronal Injury Induced by Hepatic Ischemia-Reperfusion Injury through Activating Pyroptosis in Rats

**DOI:** 10.1155/2019/3753485

**Published:** 2019-11-13

**Authors:** Limei Zhang, Hanyu Liu, Lili Jia, Jingshu Lyu, Ying Sun, Hongli Yu, Hongxia Li, Weihua Liu, Yiqi Weng, Wenli Yu

**Affiliations:** ^1^Tianjin Medical University First Center Clinical College, Tianjin 300070, China; ^2^Department of Anesthesiology, Tianjin First Center Hospital, Tianjin 300192, China

## Abstract

**Background:**

The neuronal injury and cognitive dysfunction after liver transplantation have severe effects on the prognosis and life quality of patients. Accumulating evidence suggests that both exosomes and pyroptosis could participate in hepatic ischemia-reperfusion injury (HIRI) and play key roles in neuronal death. However, the link between exosomes and neuronal pyroptosis in HIRI awaits further investigation.

**Methods:**

After establishing the HIRI rat models, we primarily studied the role of pyroptosis in hippocampal and cortical neuron injury through detecting NOD-like receptor protein 3 (NLRP3), pro-caspase-1, cleaved-caspase-1, apoptosis-associated speck-like protein containing CARD (ASC), gasdermin D (GSDMD), interleukin-1beta (IL-1*β*), and interleukin-18 (IL-18) expressions with western blotting, immunohistochemical staining, and enzyme-linked immunosorbent assay (ELISA). Then, we intravenously injected normal male rats with exosomes isolated from the sera of HIRI-challenged rats and pretreated rats with MCC950, a specific inhibitor of NLRP3, and carried out the same assay. We also detected the levels of reactive oxygen species (ROS), superoxide dismutase (SOD), and malondialdehyde (MDA) in the hippocampal and cortical tissues.

**Results:**

The results indicated that NLRP3 inflammasome and caspase-1-dependent pyroptosis were activated in the hippocampus and cortex of HIRI rats. Furthermore, serum-derived exosomes from HIRI-challenged rats not only had the ability to cross the blood-brain barrier (BBB) but also had the similar effects on neuronal pyroptosis. Moreover, ROS and MDA productions were induced in the HIRI and exosome-challenged groups. In addition, to some degree, MCC950 could alleviate HIRI-mediated hippocampal and cortical neuronal pyroptosis.

**Conclusion:**

This study experimentally demonstrated that circulating exosomes play a critical role in HIRI-mediated hippocampal and cortical injury through regulating neuronal pyroptosis.

## 1. Introduction

Hepatic ischemia-reperfusion injury (HIRI) is an important pathophysiological process in many liver operations. Hepatic ischemia-reperfusion can promote the release of cytokines, chemokines, and ROS, inducing inflammation and oxidative stress reaction, and result in cell death (apoptosis, necrosis, autophagy, etc.) leading to the injury of the liver and distant organs [1, 2]. Moreover, a recent study demonstrated that the average incidence of neurological complications after liver transplantation is about 20% [3], which severely affect the prognosis of patients and reduce their quality of life. However, the underlying mechanism of neuronal injury secondary to HIRI has not yet been completely elucidated.

Pyroptosis, a programmed inflammatory necrotic cell death, is characterized by the maturation of caspase-1, which ultimately leads to the cleavage of the pore-forming protein GSDMD, formation of cytotoxic pores in the cell membrane [4], and release of cytokines IL-1*β* and IL-18 [5]. Increasing evidence has confirmed that the activation of inflammasomes is responsible for the development of pyroptosis [6] and the NLRP3 inflammasome is the most important and representative one. The cytosolic pattern recognition receptors (PRRs), such as NLRP3, which detected the cellular pathogen-associated molecular patterns (PAMPs) and damage-associated molecular patterns (DAMPs), assemble with ASC, and then the complex can recruit and activate pro-caspase-1 through autoproteolysis leading to the cleavage and release of GSDMD, IL-1*β*, and IL-18 and subsequent inflammatory response [7]. Researchers have demonstrated that cerebral ischemia-reperfusion injury could induce astrocytic cell pyroptosis leading to neurological deficits in vivo and vitro [8]. Recent studies suggest that NLRP3 inflammasome and cleaved-caspase-1 expression were upregulated in brain diseases [9, 10]. However, whether NLRP3 inflammasome and pyroptosis mediate the neuron injury in HIRI remains unclear.

Exosomes, approximately 30-100 nm in size, are a group of circular or elliptical membrane vesicles secreted by various cells [11], which carry many functional components: lipids, protein, mRNA, microRNA, and so on [12]. They act as mediators in intercellular information transmission and cell-to-cell communication through blood circulation [13, 14]. Accumulating evidence suggests that exosomes have the ability to cross the blood-brain barrier (BBB) and play an important role in neurological diseases, especially in neuroinflammation and neurodegeneration [15–17]. The previous experimental study has shown that circulating exosomes may act as a neuroinflammatory mediator in systemic inflammation [15]. Therefore, whether exosomes modulate HIRI-induced neuronal injury awaits further study.

Based on the above findings, we aimed to investigate whether or not pyroptosis occurred in HIRI-induced neuronal injury and the role of exosomes in this pathologic process.

## 2. Methods and Materials

### 2.1. Experimental Design

Healthy, male Sprague-Dawley rats (6 weeks or so) bought from the experimental animal center of Spyford Biotechnology Incorporated Company (Beijing, China), weighing 180–200 g, were housed and bred in the standard animal care facility under a 12/12 h light/dark cycle with ad libitum access to food and water. The temperature was controlled at 23–24°C. All animal experiments were performed complying with the National Institute of Health (NIH) guidelines regarding animal experimentation and were approved by the Ethics Committee of Tianjin First Center Hospital (Tianjin, China).

To explore whether HIRI induces neuronal pyroptosis and its time-dependent relation, forty-eight rats were randomly allocated to two groups: the sham group (group S, *n* = 8) and the hepatic ischemia-reperfusion group (group IR, 90 min of ischemia followed by different durations of reperfusion: 2 h, 6 h, 24 h, 3d, and 7d; *n* = 8/group) for enzyme-linked immunosorbent assay, western blotting, and the levels of oxidative stress. To determine the role of exosomes, fifteen rats were randomly assigned to three groups: the sham group (group S', *n* = 5), the IR group (group IR', *n* = 5), and the exosome group (group EXO, *n* = 5). Group S' and group IR' were injected with 100 *μ*l PBS via the tail vein before the operation, and group EXO was subjected to exosomes, which were purified from 2.5 ml sera of group IR, dissolved in 100 *μ*l PBS. Furthermore, twenty-eight rats were randomly divided into five groups for histology: group S (*n* = 5), group IR (*n* = 5), group S' (*n* = 5), group IR' (*n* = 5), and group EXO (*n* = 8, three of them are used in determining exosomes' ability to cross BBB). In addition, to confirm the role of NLRP3 inflammasome, thirty rats were randomly allocated to six groups: the sham operation group pretreated with normal saline (NS) (group N, *n* = 5), the sham operation group pretreated with MCC950 (group M, *n* = 5), the IR group pretreated with NS (group IR+N, *n* = 5), the IR group pretreated with MCC950 (group IR+M, *n* = 5), the exosome group pretreated with NS (group E+N, *n* = 5), and the exosome group pretreated with MCC950 (group E+M, *n* = 5). In our pilot study, we experimented three gradient doses (10 mg/kg, 30 mg/kg, and 50 mg/kg) of MCC950 and injected intraperitoneally 2 hours before modeling. MCC950 (Selleckchem, USA) was diluted to 10 mg/ml with NS before injection [18].

### 2.2. Animal Model

The 70% warm HIRI model was carried out as previously described [19, 20]. In brief, the rats were fasted for 12 h before operation without limiting water. All animals were anesthetized with 1% amyl sodium pentobarbital (30 mg/kg, intraperitoneally), which were monitored through observing the color of the lip mucosa and the movement of the thorax. The intestines were exteriorized by a 3 cm midline abdominal incision to expose hepatic portal, and then, the left hepatic artery and portal vein were clamped with a microvascular clip, which accounted for 70% of the total liver in rat approximately. After 90 minutes of ischemia, the clip was removed and the wound was closed by sterile suture after the abdominal cavity was rinsed with 0.9% NS. A heat lamp was used to keep body temperature around 37°C, and pulling the tongue out, oxygen uptake and the color of the lip mucosa and the thoracic fluctuation were closely monitored to raise survival rate. In the sham group, we separated the vessels and bile duct pedicles but these are not clamped. At the end of the experiment, the whole blood was collected through the inferior cava vein and then rats were intracardially perfused with phosphate-buffered saline (50 mM PBS, pH = 7.4) under deep anesthesia. The blood was centrifuged at 3000 × g for 15 min after sitting undisturbed at room temperature for 30 min to separate the serum. Brain samples were carefully harvested after decapitating and opening the cranium, washed with cold NS, and used for subsequent experimental procedures.

### 2.3. Measurement of ROS, SOD, and MDA Levels

The levels of ROS, SOD, and MDA in the hippocampal and cortical tissues were detected using a corresponding assay kit (Nanjing Jiancheng Corp., Nanjing, China) according to the manufacturer's instructions.

### 2.4. Exosome Isolation, Recognition, Protein Extraction, and Labeling

Exosomes were extracted from the sera using the ExoQuick serum exosome precipitation solution in accordance with the manufacturer's instructions (EXOQ5A-1, Systems Biosciences, San Francisco, CA, USA). For differential ultracentrifugation, the serum samples were centrifuged at 20, 000 × g at 4°C for 30 min to remove cell debris and filtered with a 0.22 *μ*m filter to remove large vesicles and then were centrifuged at 110,000×g at 4°C for 90 min. The pellets, enriched exosomes, were resuspended in 1× PBS. The suspension was centrifuged again at 110, 000 × g at 4°C for 90 min. Exosomes purified by ultracentrifugation were used for injection in vivo, and a detection under the transmission electron microscope. Exosome protein extraction was performed as previously described [15]. In brief, the exosomes isolated from the same volume of serum sample were resuspended in PBS (1/8 volume of the input serum) and were mixed with radioimmunoprecipitation (RIPA) buffer in a portion of 1 : 1, then centrifuged at 13000 × g at 4°C for 20 min. The supernatant of equal volume of each experimental group was taken and added to the equal volume of 5 × loading buffer (1/4 volume of supernatant), and then, the protein was denatured by heating at 100°C for 10 min. The transmission electron microscope was used to determine the size of exosomes. Exosomes were labeled with PKH26 complying with the manufacturer's instructions (PKH26 Red Fluorescent Cell Linker Mini Kits, Sigma-Aldrich, St. Louis, MO, USA) and were detected by immunofluorescence microscopy.

### 2.5. Immunohistochemical Staining

Brains were collected quickly as described above and then were fixed with 10% formaldehyde and embedded by paraffin. A microtome was used to make 4 *μ*m thick slices. Slices were dewaxed thrice with xylene, then rehydrated in a graded series of ethanol (100%, 95%, and 70%, diluted in distilled water) and in distilled water. Heat-induced epitope retrieval was performed at 95-99°C in citrate buffer (pH = 6.0) for 18 minutes. The slides were treated with 3% hydrogen peroxide to eliminate human peroxidase activity, blocked with 10% normal goat serum (Zsbio, China), and stained using the NLRP3 antibody (1 : 200, Abcam, USA) diluted by antibody diluent (Zsbio, China), using the Envision System HRP DAB (Zsbio, China). The cell nucleus was counterstained using hematoxylin. Images were acquired within the hippocampal and cortical regions with an Olympus microscope.

### 2.6. Western Blotting

Each frozen hippocampal and cortical tissue (weighing 30-50 mg) and precooled RIPA lysis buffer (Solarbio, China), added with protein phosphatase inhibitor complex (Biomed, China) and phenylmethylsulfonyl fluoride (PMSF, BestBio, China), were mixed in a proportion of 1 : 5, ground into homogenate, and centrifuged for liquid supernatant. The supernatant was then quantified by Pierce™ BCA protein assay (23225, Thermo Fisher Scientific, Waltham, MA, USA). Protein samples were separated with 12% SDS-PAGE gel and transferred onto PVDF (Millipore, USA) membranes. The membranes were incubated with antibodies of NLRP3 (1 : 1000, Abcam, USA), pro-caspase-1 (1 : 500, Wanleibio, China), cleaved-caspase-1 (1 : 500, Wanleibio, China), ASC (1 : 1000, Abcam, USA), GSDMD (1 : 1000, Abcam, USA), CD63 (1 : 500, Wanleibio, China), CD9 (1 : 1000, Abcam, USA), and GAPDH (1 : 1000, Cell Signaling Technology, USA), respectively, overnight at 4°C. After washing by TBST, the membranes were incubated with horseradish enzyme labeling goat anti-rabbit IgG (1 : 5000, Boster, China) for 1 h at room temperature and subjected to ECL detection reagent (Millipore, USA). The protein bands were visualized by autoradiography and analyzed by ImageJ.

### 2.7. Determination of the Levels of IL-1*β* and IL-18 in Serum

The levels of IL-1*β* and IL-18 in serum were measured using enzyme-linked immunosorbent assay (ELISA) kit (Shanghai Biovol Corp., Shanghai, China) in compliance with the manufacturer's instructions.

### 2.8. Statistics

Statistical analysis was carried out using SPSS (version 22.0; SPSS for Windows, Chicago, IL) software. Quantitative values were expressed as the mean ± SD and analyzed using one-way analysis of variance (ANOVA). A *p* value of less than 0.05 was considered statistically significant.

## 3. Results

### 3.1. HIRI Led to Hippocampus and Cortex Oxidative Stress Reaction

**Figure 1:**
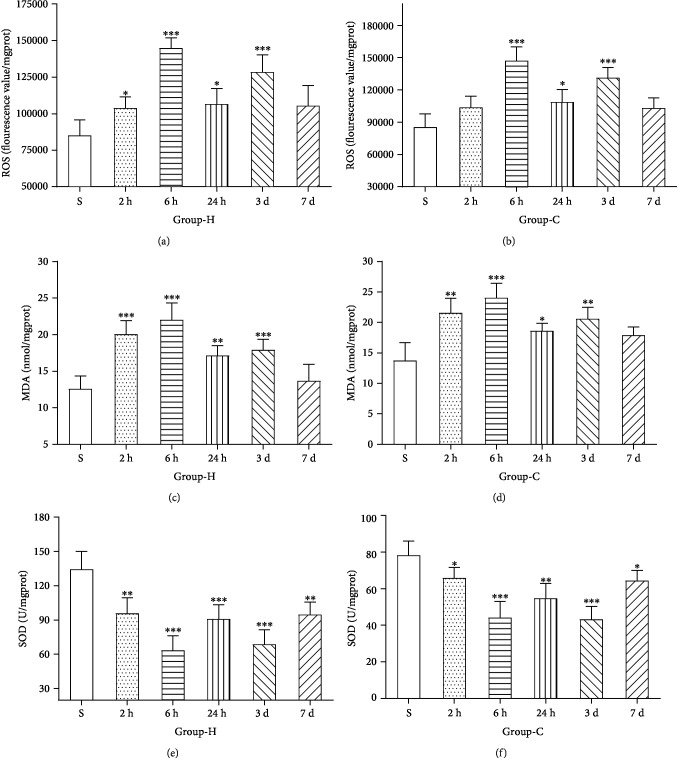
Quantitative ELISA results of hippocampal and cortical ROS, MDA, and SOD after inducing HIRI or, respectively, after sham operation. (a, b) The levels of ROS in the hippocampus (H) and cortex (C). (c, d) The levels of MDA in the hippocampus (H) and cortex (C). (e, f) The levels of SOD in the hippocampus (H) and cortex (C). *n* = 8 per group. Data are presented as the mean ± SD. ^∗^*p* < 0.05 vs. the sham group; ^∗∗^*p* < 0.01 vs. the sham group; ^∗∗∗^*p* < 0.0001 vs. the sham group.

ROS, SOD, and MDA were detected to define the level of the hippocampus and cortex oxidative stress under the HIRI model. Compared with group S, the levels of ROS and MDA were significantly increased with a time-dependent manner in the hippocampus and cortex in group IR (*p* < 0.05), as shown in Figures 1(a)–1(d). Nevertheless, compared with group S, the activity of SOD in the hippocampus and cortex showed a marked decrease in group IR (*p* < 0.05), as shown in Figures 1(e) and 1(f). All the results proved that HIRI increased the levels of oxidative stress in hippocampal and cortical tissues.

### 3.2. HIRI-Induced Hippocampal and Cortical Tissues NLRP3 Inflammasome Activation and Pyroptosis in a Time-Dependent Manner

**Figure 2:**
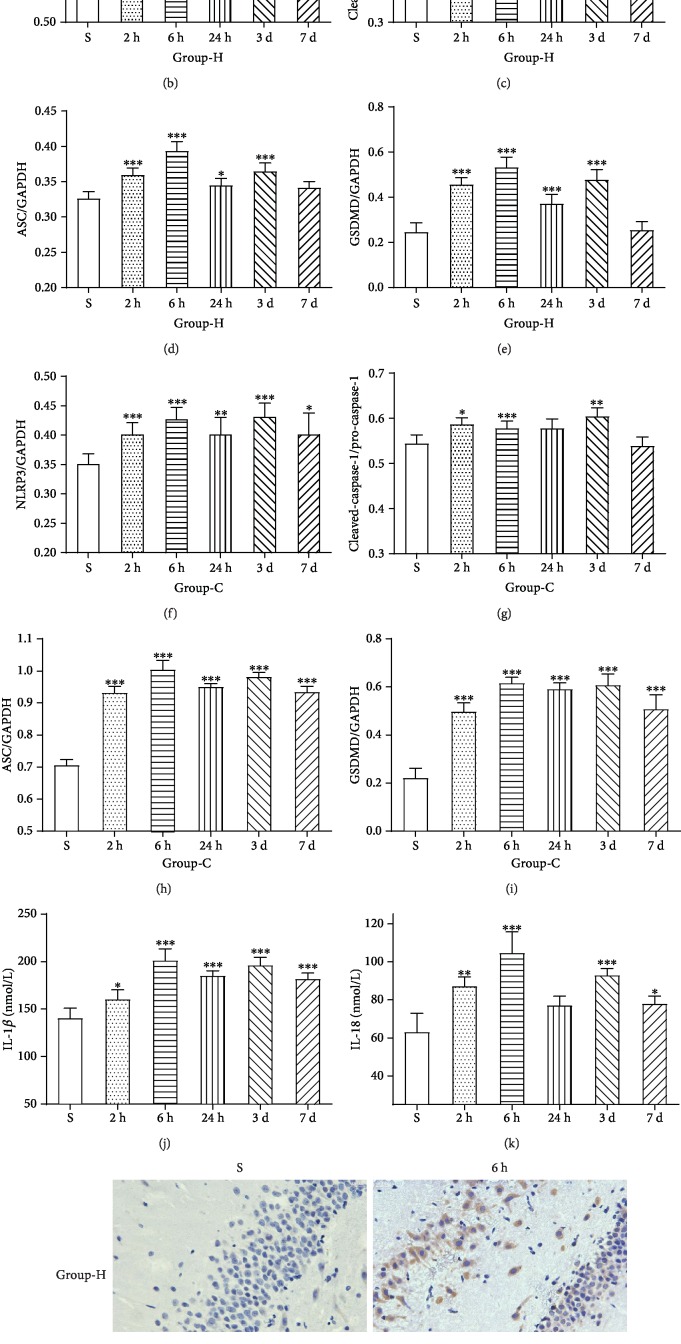
HIRI induces hippocampal (H) and cortical (C) tissue pyroptosis in a time-dependent manner. Western blotting assays of hippocampal and cortical tissues exposed to HIRI for the detection of activity levels of NLRP3 (NOD-like receptor protein 3, 114 kDa), pro-caspase-1 (the precursor form, 35-45 kDa), cleaved-caspase-1 (the active form, 20 kDa), ASC (apoptosis-associated speck-like protein containing CARD, 33 kDa), and GSDMD (gasdermin-D, 53 kDa). In addition, GAPDH (glyceraldehyde 3-phosphate dehydrogenase, 37 kDa) is used as the loading control. (a–i) Representative images and quantitative photometric analyses of western blotting demonstrating the pyroptosis-related protein of the hippocampus and cortex for each experimental group. (j, k) Quantitative analyses of the ELISA results of IL-1*β* and IL-18 (the products of pyroptosis). *n* = 8 per group. Data are presented as the mean ± SD. ^∗^*p* < 0.05 vs. the sham group; ^∗∗^*p* < 0.01 vs. the sham group; ^∗∗∗^*p* < 0.0001 vs. the sham group. (l) Representative images of immunohistochemical staining with NLRP3 antibody in the hippocampus and cortex (magnification, ×400).

Western blotting, immunohistochemical staining, and enzyme-linked immunosorbent assay were used to determine the expression of NLRP3 inflammasome and neuronal pyroptosis in hippocampal and cortical tissues. The expressions of NLRP3, cleaved-caspase-1, ASC, and GSDMD were increased in group IR compared with group S as shown in Figures 2(a)–2(i) (*p* < 0.05), which all reached the peak at 6 hrs after reperfusion and hit the minimum at 7 d reperfusion duration. Furthermore, the maturation of IL-1*β* and IL-18 was consistent with this trend (Figures 2(j) and 2(k), *p* < 0.05). After this experiment, 6 hrs of duration for reperfusion, the peak time point, was adopted for the rest of all HIRI models. The histological results also showed that hippocampal and cortical NLRP3 protein expression in group IR, 6 hrs after reperfusion, was more evident than group S (Figure 2(l), *p* < 0.05). These facts drop a hint that pyroptosis and NLRP3 inflammasome were imperative to HIRI-induced hippocampal and cortical injury.

### 3.3. HIRI Increased the Levels of Exosomes in Serum

**Figure 3:**
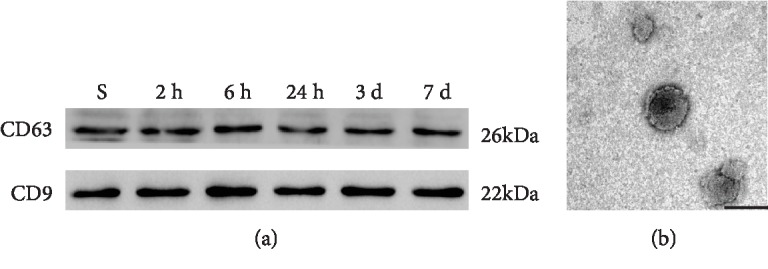
The determination of extracted exosomes. (a) Representative images of western blotting of CD63 and CD9 (marker protein of exosomes) for each experimental group. (b) Representative photomicrograph of exosomes detected by a transmission electron microscope. Scale bar: 100 nm.

The same volume serum of every sample (400 *μ*l) was used for purifying exosomes, and then exosomes were resuspended in 50 *μ*l PBS individually. The protein extraction was performed as described before. The isometric supernatant of each sample was mixed with the same volume of loading buffer (30 *μ*l). The protein samples were diffused by SDS-PAGE gel and detected with CD63 and CD9. The results are shown in Figure 3(a), and the expressions of CD63 and CD9 were higher in group IR at different reperfusion durations compared with group S and both peaked in 2 hrs after reperfusion. Furthermore, the ultra-microstructure of exosomes was observed to clear and define their existence under a transmission electron microscope (Figure 3(b)). Predicated on these findings, the HIRI could promote the production of exosomes.

### 3.4. Exosomes Crossed the BBB

**Figure 4:**
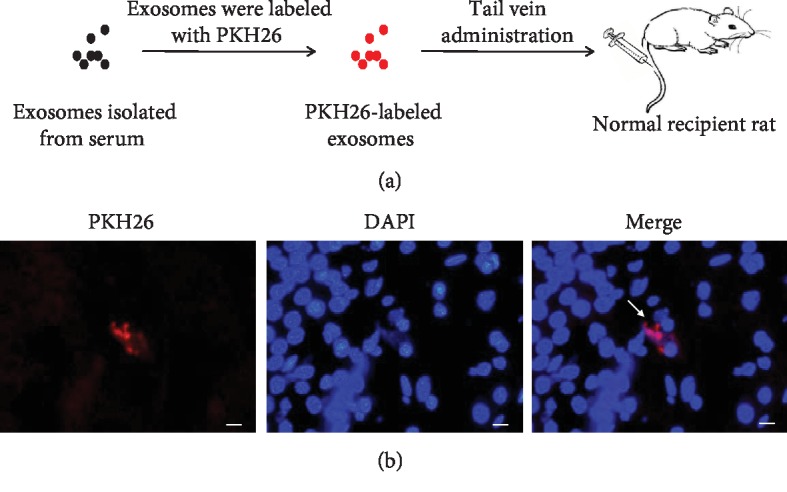
Intake of intravenously injected PKH26-labeled exosomes in the brain. (a) A flow chart of the experimental procedure showed the process of labeling exosomes with PKH26 and then injection in vivo. Exosomes purified from the serum were labeled with PKH26, exhibiting the red fluorescence, and administered intravenously to the recipient rat. (b) Representative images of the brain sections of the recipient rat. PKH26-labeled exosomes were shown in red, and DAPI-labeled nucleuses were shown in blue. White arrowheads indicated PKH26-labeled exosomes (magnification, ×400).

As shown in Figure 4(a), to testify that the exosomes could go through the blood-brain barrier, exosomes, labeled by PKH26, were administrated to the normal rats via tail vein and the brain tissues' frozen slices were detected by the immunofluorescence microscope. As shown in Figure 4(b), the red fluorescent markers were clustered around the blue nucleus in the brain tissue, which proved that exosomes could cross the BBB and enter the brain tissue undoubtedly.

### 3.5. Exosomes Isolated from Sera of HIRI Rat Model Could Partly Lead to Oxidative Stress and Hippocampal and Cortical Neuronal Injury

**Figure 5:**
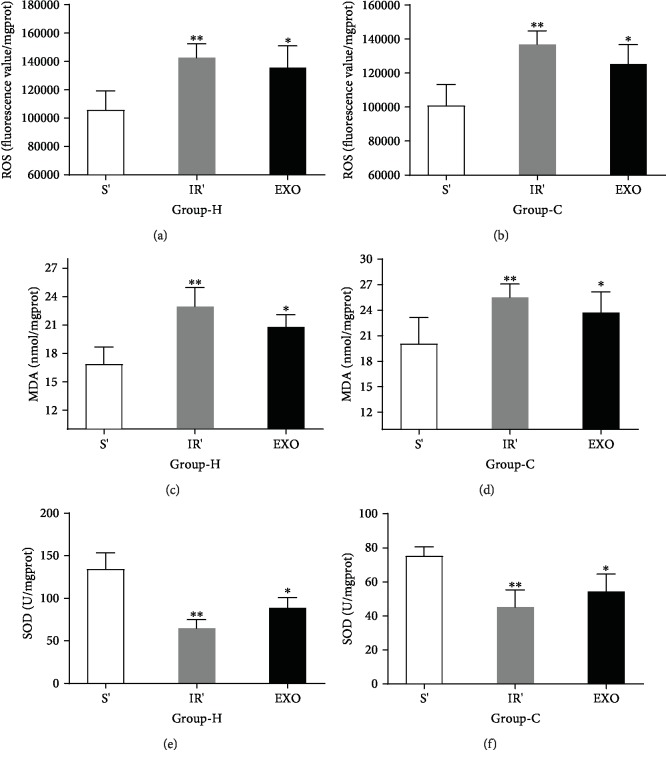
Exosomes isolated from sera of HIRI rat could partly lead to oxidative stress response. (a–f) The levels of ROS, MDA, and SOD in the hippocampus (H) and cortex (C). *n* = 5 per group. Data are presented as the mean ± SD. ^∗^*p* < 0.05 vs. the sham group; ^∗∗^*p* < 0.01 vs. the sham group; ^∗∗∗^*p* < 0.0001 vs. the sham group.

**Figure 6:**
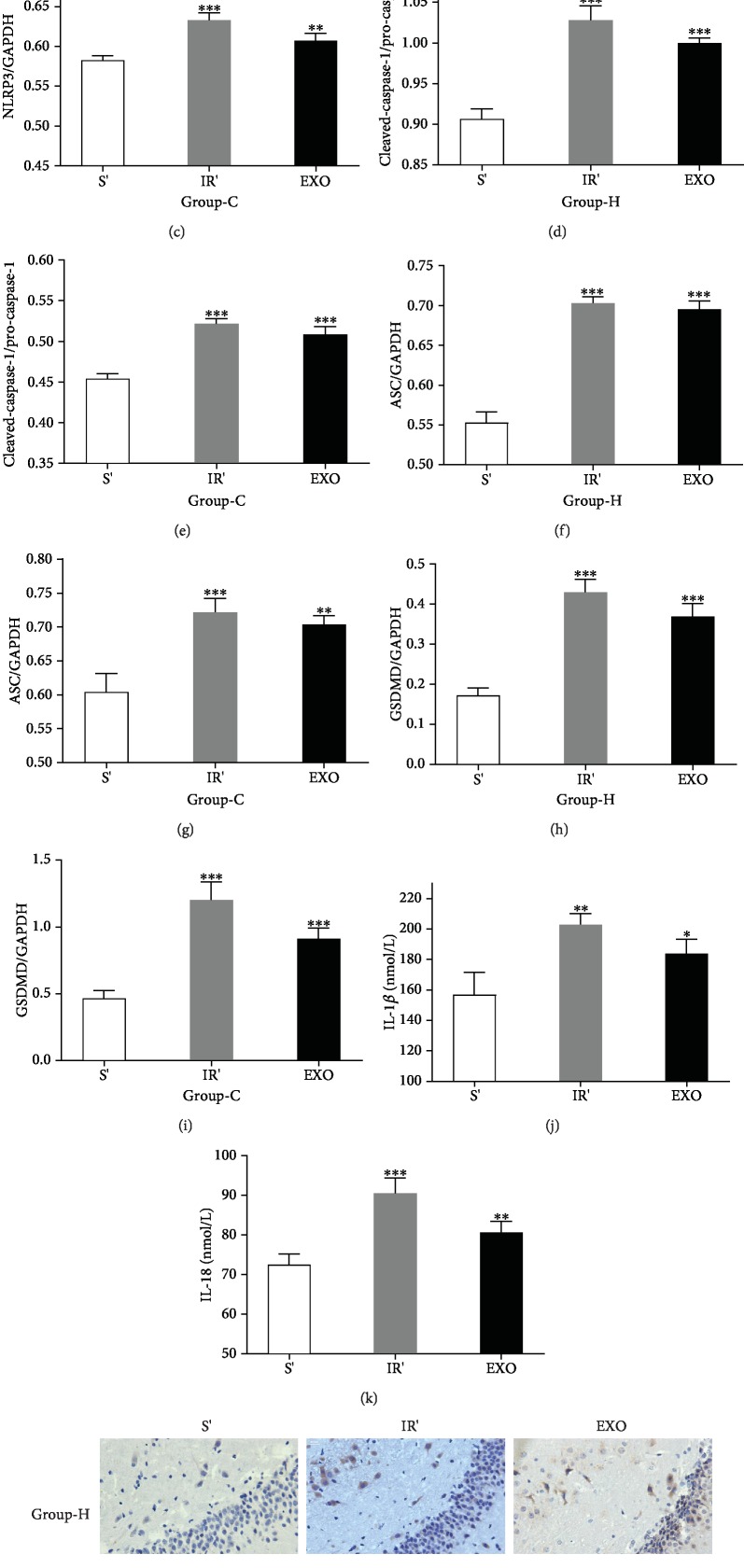
Exosomes isolated from sera of HIRI rat could partly lead to hippocampal (H) and cortical (C) neuronal injury. (a) Representative images of western blotting demonstrating the pyroptosis-related protein of the hippocampus and cortex for each experimental group. (b–i) Quantitative photometric analyses of western blotting illustrate the mostly significant upregulation of pyroptosis-related protein after HIRI and HIR exosome pretreatment. (j, k) Quantitative analyses of the ELISA results of IL-1*β* and IL-18 (the products of pyroptosis). (l) Representative images of immunohistochemical staining with NLRP3 antibody in the hippocampus and cortex (magnification, ×400). *n* = 5 per group. Data are presented as the mean ± SD. ^∗^*p* < 0.05 vs. the sham group; ^∗∗^*p* < 0.01 vs. the sham group; ^∗∗∗^*p* < 0.0001 vs. the sham group.

As shown in Figures 5(a)–5(f), the levels of oxidative stress response were measured and it was found that compared with those in group S', ROS and MDA were higher in group IR' and group EXO, whereas SOD was decreased in group IR' and group EXO (*p* < 0.05). The NLRP3, cleaved-caspase-1, ASC, and GSDMD expressions in the hippocampus and cortex increased in group IR' and group EXO compared to group S' (Figures 6(a)–6(i), *p* < 0.05). The levels of IL-1*β* and IL-18 had a similar tendency to the protein expression (Figures 6(j) and 6(k), *p* < 0.05). What is more, the immunohistochemical results (Figure 6(l), *p* < 0.05) reconfirmed the same fact that exosomes played a partial role in HIRI-induced hippocampal and cortical neuronal pyroptosis.

### 3.6. MCC950 Could Attenuate Hippocampal and Cortical Neuronal Injury Associated with HIRI and HIRI-Derived Exosomes

**Figure 7:**
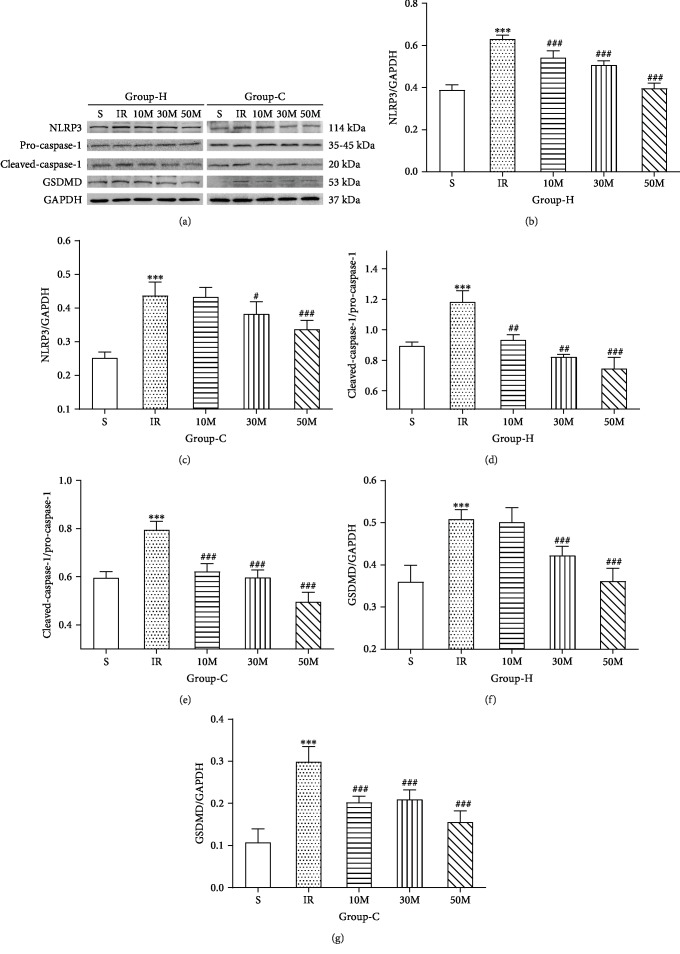
The more effective dosage of MCC950, the specific inhibitor of NLRP3, for inhibiting hippocampal (H) and cortical (C) neuronal pyroptosis induced by HIRI. (a) Representative images of western blotting demonstrating the pyroptosis-related protein of the hippocampus and cortex for each experimental group. (b–g) Quantitative photometric analyses of western blotting illustrate the pyroptosis-related protein of the hippocampus and cortex for each experimental group. *n* = 5 per group. Data are presented as the mean ± SD. ^∗^*p* < 0.05 vs. the sham group; ^∗∗^*p* < 0.01 vs. the sham group; ^∗∗∗^*p* < 0.0001 vs. the sham group; ^#^*p* < 0.05 vs. the IR group; ^##^*p* < 0.01 vs. the IR group; ^###^*p* < 0.0001 vs. the IR group.

To investigate the effective dose of MCC950, the small molecular specific inhibitor of NLRP3, we employed three gradient doses (10 mg/kg, 30 mg/kg, and 50 mg/kg) in our preliminary experiment and measured expression levels of NLRP3, pro-caspase-1, cleaved-caspase-1, and GSDMD in brain tissues. As shown in Figures 7(a)–7(g), compared with group IR+N, the levels of NLRP3 inflammasome-associated protein gradually decreased in the MCC950 pretreatment groups and the group of 50 mg/kg exhibited the strongest inhibitory effect, whereas the sham groups showed no significant influences. Therefore, we all adopted the dose of 50 mg/kg in our subsequent experiment.

**Figure 8:**
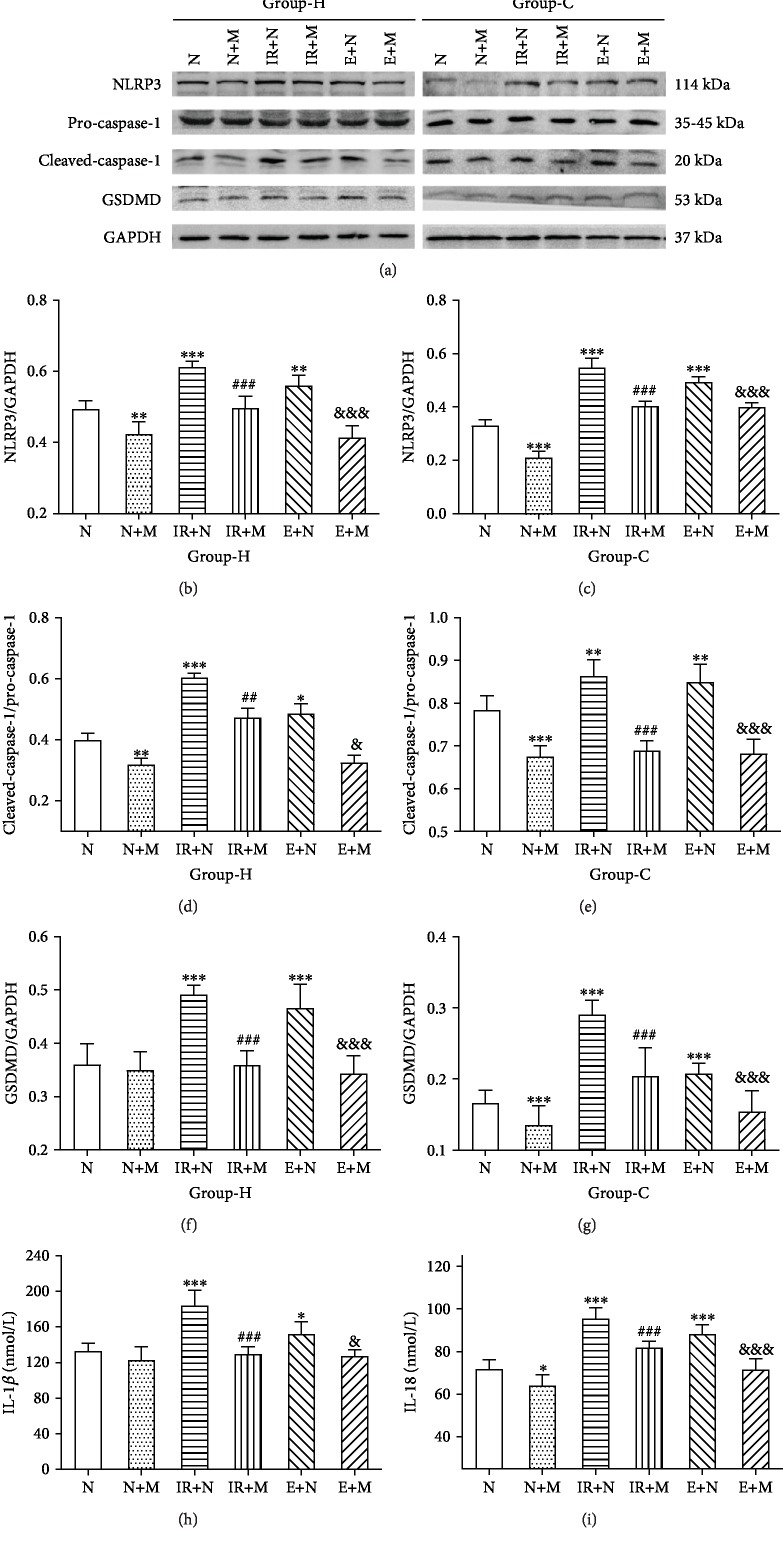
MCC950 could partly attenuate hippocampal (H) and cortical (C) neuronal injury associated with HIRI and HIRI-derived exosomes. (a) Representative images of western blotting demonstrating the pyroptosis-related protein of the hippocampus and cortex for each experimental group. (b–g) Quantitative photometric analyses of western blotting demonstrating the mostly upregulated pyroptosis-related protein of the hippocampus and cortex for each experimental group. (h, i) Quantitative analyses of the ELISA results of IL-1*β* and IL-18. *n* = 5 per group. Data are presented as the mean ± SD. ^∗^*p* < 0.05 vs. the N group; ^∗∗^*p* < 0.01 vs. the N group; ^∗∗∗^*p* < 0.0001 vs. the N group; ^#^*p* < 0.05 vs. the IR+N group; ^##^*p* < 0.01 vs. the IR+N group; ^###^*p* < 0.0001 vs. the IR+N group; ^&^*p* < 0.05 vs. the E+N group^; &&^*p* < 0.01 vs. the E+N group; ^&&&^*p* < 0.0001 vs. the E+N group.

To determinate the role of the NLRP3 inflammasome in hippocampal and cortical neuronal injury, we pretreated rats with MCC950 before molding. Comparing the results of the MCC950 pretreatment group to those of the vehicle pretreatment group, NLRP3 and its related products showed an even more distinct and significant decrease, both after inducing HIRI and after HIRI-derived exosome administration (Figures 8(a)–8(g), *p* < 0.05). In addition, distinct decreases in serum levels of IL-1*β* and IL-18 could be found between these groups (Figures 8(h) and 8(i), *p* < 0.05). Therefore, MCC950 could partly alleviate hippocampal and cortical neuronal pyroptosis induced by HIRI and HIRI-derived exosomes.

## 4. Discussion

It is well known that operation is the most effective curative treatment for end-stage liver diseases. The temporary interruption of liver blood perfusion is the most important procedure in many liver-related operations, and HIRI is the main pathophysiological process involving both inflammatory and oxidative stress responses [21, 22]. The generated ROS, cytokine, and other inflammatory factors were the main stimuli for the liver and distant organs. The brain is one of them, and HIRI-related neurological complication includes encephalopathy, epileptic seizures, posterior leukoencephalopathy syndrome, infection, and central pontine myelinolysis [23]. These complications are closely associated with significant mortality and morbidity and lead to a longer duration of postoperative hospitalization [24]. In addition, the incidence of neurological complication after pediatric liver transplantation, which resulted from biliary atresia and other protopathy, is as high as 46% due to the vulnerability of a developmental brain [25]. Recently, growing evidence showed that HIRI could even lead to short-term or long-term cognitive impairment [26]. Therefore, mechanisms and strategies to minimize the negative effects of brain injury induced by HIRI are now the focus of clinical and experimental studies.

Much of the published literature has focused on the influence of classical inflammatory pathways, for example, the JAK2/STAT3 pathway [27] and NF-*κ*B pathway [26]. Our recent research has unraveled that N-methyl-D-aspartate (NMDA) receptor subunit 2A (NR2A) played a critical role in HIRI-related hippocampal injury and long-term cognitive dysfunction through the Src-PSD95-NR2A pathway [28], and other study also proves that cognitive dysfunction induced by HIRI was associated with reduction of NR2B expression [29]. Collectively, the ultimate form of neuronal injury is almost related to neuronal apoptosis or necrosis, which is the most common type of cell death, whereas pyroptosis, a newly discovered form of cell death, is rarely considered a damage mechanism in HIRI-induced neuronal injury. Pyroptosis was beginning to be noticed in Salmonella and Shigella species infection [30, 31], starting with the formation of the inflammasomes and leading to the maturation and release of IL-1*β* and IL-18 and subsequent inflammatory cascade reaction, and was exclusively dependent on caspase-1. Nevertheless, the classical process of pyroptosis was markedly changed until 2015 when Dixit and Shao nearly simultaneously found that inflammatory caspases, caspase-1 and caspase-11, cleaved GSDMD into a 31 kDa N-terminal fragment and a 22 kDa C-terminal fragment [32, 33]. Afterwards, several landmark studies independently demonstrated that N-terminus itself could specifically bind to phosphoinositide and cardiolipin on the plasma membrane, exhibiting the pore-forming activity, and promote the release of inflammatory factors and pyroptotic cell death [34–38]. Thereby, GSDMD functions as the marker and direct executor of pyroptotic cell death. Pyroptosis plays a significant role in a variety of biological systems such as the central nervous [39, 40], immune [41], and cardiovascular systems [42, 43]. Inflammasomes, which were first described in 2002 as cytosolic caspase-activating protein complexes in macrophage lineage cells, could sense specific host or infectious stimuli (ROS, ATP, bacteria, and so on) and then activate caspase-1 [5, 44, 45]. An experimental study has suggested that inflammasome activation could aggravate the neuroinflammatory response and consequently increase central nervous system (CNS) damage in neonates with fetal neural ZIKV infection [46]. A large body of evidence has confirmed that the activation of inflammasomes in microglia, especially NLRP1 and NLRP3 inflammasomes, and pyroptotic cell death played a pivotal role in traumatic brain injury (TBI) in the CNS [39, 47, 48]. Besides, accumulating studies of cerebral stroke, ischemia, or hemorrhagic have reported that the selective inhibitor of caspase-1, AC-YVAD-CMK, was proved to have a therapeutic effect and pyroptosis was involved in the pathophysiological process [40, 49–51]. A previously published study found that the activity of cells in specific brain structures, anatomically or functionally associated with the liver, was reflected by Fos immunoreactivity and showed an increase under HIRI condition [52]. Besides, IL-1*β* administration could also increase neuronal activations, which was showed by Fos immunoreactivity [53]. What is more, recent studies have identified that specific inhibition of IL-1*β*, both in mice pretreated with IL-1 receptor antagonist and in IL-1R^–/–^ mice, alleviated the neuroinflammatory effects of operation and memory impairment [54]. Hence, these facts drop us a hint that pyroptosis is involved in neuroinflammation and neuronal injury in the CNS. Our present data showed that the trends of NLRP3, cleaved-caspase-1, ASC, and GSDMD are concordant, begun to increase at 2 hrs, peaked at 6 hrs, slightly decreased at 24 hrs, rose to another peak at 3 days after reperfusion, and then reduced again. This trend may be interpreted as the initial damage which was due to the entry of HIRI-derived harmful substances into brain tissues, may hit the peak at 6 hrs, and may be reduced at 24 hrs after reperfusion, and then hippocampus and cortex pyroptosis-induced subsequent inflammatory cascade reaction resulted in secondary brain injury, which peaked again at 3 days after reperfusion.

The fact that oxidative stress is a major pathophysiologic mechanism during HIRI has been recognized for many years. Furthermore, previous studies demonstrated that ROS has been proposed as the trigger for NLRP3 inflammasome activation [55, 56] and mitochondrial ROS scavengers could reduce brain injury through NLRP3 downregulation [57]. The NLRP3 inflammasome is the most common inflammasome in the CNS and one of the dominant contributors to neuroinflammation [58]. Therefore, we postulated that HIRI could contribute to the neuronal injury through hippocampal and cortical NLRP3 inflammasome activation and neuron pyroptosis. MCC950 is a small molecular and selective inhibitor of NLRP3 inflammasome and capable of inhibiting NLRP3 inflammasome formation, specifically reducing pyroptotic cell death and IL-1*β* signaling [18]. In that context, we speculated that MCC950 may attenuate hippocampal and cortical injury induced by HIRI, and our results aligned with the hypothesis.

However, there are many questions remaining—how the liver and brain were linked, by the direct harmful products or other mediators or maybe both?

Exosome, a nanoparticle extracellular vesicle, possesses a phospholipid bilayer membrane and can transport various kinds of contents to recipient cells. The latest studies have indicated that exosomes contribute to cell-cell communication and are considered closely related to the modulation of angiogenesis and neurogenesis in many neurological diseases [59]. Recent studies have identified that circulating exosomes might act as a neuroinflammatory mediator, which could provoke significant microgliosis and to a lesser extent astrogliosis, in systemic inflammation, and exosomes could go through BBB freely [15]. Moreover, leukemia-derived exosomes could play critical roles by directly modulating the integrity of endothelial cell-cell junction in vivo and pave the way for entry into the brain [60]. These facts drop us a hint that exosomes can act as information media between peripheral circulation and the CNS. A number of empirical studies have demonstrated that extracellular vesicles (EV) such as exosomes contain inflammasome protein that leads to the spread of inflammation after brain trauma, spinal cord injury [61], and stroke [62, 63]. Predicated on these facts, we postulated that exosomes could load the information of HIRI in some specific way, function as a mediator, and then transfer to brain tissues. To testify our deduction, we purified the circulating exosomes from an HIRI rat and intravenously injected them to a normal recipient rat, unveiling that exosomes could play a similar role as HIRI and contribute to brain injury and oxidative stress. However, our study solely demonstrated that exosomes could lead to neuronal pyroptotic cell death through activating NLRP3 inflammasome to some degree and the underlying mechanisms of this crosstalk are still unknown and await further investigation. Nevertheless, there are rare researches involving this crosstalk until now. A recently published study found that manganese-stimulated exosomes that contained the inflammasome component ASC propagated NLRP3 inflammasome activation in microglial cells [64]. In addition, Wang and his colleagues revealed that 22 upregulated proteins of LPS-induced macrophage-derived exosomes are involved in the NOD-like receptor signaling pathway through Kyoto Encyclopedia of Genes and Genomes analysis [65]. Interestingly, there are some researches suggesting that the phospholipids on the extracellular vesicle surface could be oxidized by ROS, could be released under the condition of HIRI, and could then lead to neutrophil activation and aggravated subsequent hepatic injury through Toll-like receptor 4 (TLR4) [66]. It is of course well established that in the CNS, microglia function as macrophages and neutrophils in the peripheral circulation. Furthermore, macrophages and neutrophils are the major sources of ROS in inflammation [67, 68]. A previous study revealed that once discharged from endothelial cells of BBB, circulating EVs can bind to leukocytes and influence their activity [69]. And several independent studies have proved that TLR4 is associated with NLRP3 inflammasome activation [70, 71]. What is more, oxidized phospholipids (OxPL), generated from the plasma membrane and circulating lipoproteins, could induce inflammatory pain through mediating transient receptor potential (TRP) channel activation [72] and lead to myocardial ischemia-reperfusion injury through the Bcl-2 death protein 3 (Bnip3) pathway [73]. We can conclude that OxPL not only is related to exosome-induced ischemia-reperfusion injury but also has a close relationship with neuronal inflammation. In that context, we postulate that the phospholipids on the exosome surface are oxidized under the condition of HIRI, cross the BBB, and lead to NLRP3 inflammasome and microglia activation through combining with TLR4. This is also the research work we are currently working on, whereas the conclusion is still unknown. Therefore, there are many questions remaining about the underlying mechanisms and awaiting further study.

Referring to previous research papers [19, 20], we succeeded in finding the 70% warm HIRI rat model and the survival rate was 100%. To mimic the HIRI with the injection of exosomes, we chose 2 ml serum of each model as the input, nearly the whole sera of one rat. The major results are concluded as follows: (1) HIRI could induce distant organ injury including the hippocampus and cortex through activating NLRP3 inflammasome and neuronal pyroptosis; (2) the level of serum exosomes showed a marked increase under the condition of HIRI, and exosomes were proved to have the ability to go through BBB freely; (3) administration of exosome-derived HIRI rat sera into a normal adult male rat could also lead to hippocampal and cortical pyroptosis; (4) the oxidative stress reactions of brain tissues were activated in group IR and group EXO; (5) the selective inhibitor of NLRP3, MCC950, could effectively ameliorate expression levels of NLRP3 and neuronal pyroptosis to a lesser extent. These findings of this study implicated that exosome-derived HIRI rat models could lead to hippocampal and cortical neuronal pyroptosis, which was partly dependent on the activation of the NLRP3 inflammasome. The above results suggested that exosomes play a significant role in HIRI-induced neuronal injury.

There are also several limitations in our present study. First, our current findings have not clearly illustrated the relation between neuronal pyroptosis and cognitive dysfunction, and the role of other inflammasomes in pyroptosis remains to be further investigated. Moreover, the underlying mechanisms of exosome-mediated neuronal pyroptosis are still unknown. Finally, the detailed cell types of pyroptosis have not been clarified in our present results and await deeper study.

Collectively, the experimental study has demonstrated that HIRI causes hippocampal and cortical neuronal pyroptosis in a time-dependent manner and exosomes play a partial role in neuronal injury induced by HIRI. By exploring this mechanism, we hope to put forward new targeted drugs to alleviate exosome releasing and neuronal pyroptosis and reduce the central nervous system complications after liver transplantation.

## Data Availability

The data used to support the findings of this study are available from the corresponding author upon request.
